# Non-operative management strategy for blunt pulmonary artery pseudoaneurysm: A case report^☆^

**DOI:** 10.1016/j.tcr.2023.100954

**Published:** 2023-10-18

**Authors:** Declan A. Feery, Kevin Kuruvilla, Allison G. McNickle, Douglas R. Fraser

**Affiliations:** Division of Trauma, Kirk Kerkorian School of Medicine at University of Nevada Las Vegas, 1701 W Charleston Blvd #400, Las Vegas, NV 89102, United States of America

**Keywords:** Pulmonary artery pseudoaneurysm, Aneurysm, Blunt trauma, Motor vehicle crash, Case report

## Abstract

Traumatic pulmonary artery pseudoaneurysms (PAP) are rare findings and are often associated with penetrating trauma to the chest. We present a case of a pulmonary artery pseudoaneurysm following blunt trauma. A 49-year-old man presented after a motor vehicle collision. Contrast enhanced computed tomography scans of the neck, chest, abdomen, and pelvis were obtained demonstrating a proximal right pulmonary artery pseudoaneurysm, small volume hemopericardium, left first rib fracture, and focal non-flow limiting dissection of left subclavian artery. For the management of right PAP, we adopted a non-operative management strategy with an esmolol infusion for strict heart rate and blood pressure control. An echocardiogram was obtained the next day revealing no cardiac tamponade. Angiography of the chest was done after 24 h which showed stable appearance of the right PAP and hemopericardium. Patient was discharged home on hospital day 11.

## Introduction

Pulmonary artery (PA) aneurysms and PAPs are rare findings following traumatic injuries. The etiology of PA aneurysms varies from congenital disease, vasculitis, infection, and trauma. PAPs can be asymptomatic and are classically found incidentally on computed tomography (CT) imaging performed for other reasons, but in rare circumstances the pseudoaneurysm can be appreciated on plain film [1]. These can be lethal in the setting of rupture, cardiac tamponade, coronary artery compression, or dissection [2]. Due to the acuity of clinically significant PAP and rapid decompensation with rupture, the diagnosis is often made at autopsy [3]. Pseudoaneurysms have a higher risk of bleeding relative to true aneurysms due to the more friable aneurysmal wall, making it an important pathology to recognize [4].

PAPs can be difficult to manage given their location. There are currently no standardized management guidelines for these injuries. Several treatment options have been described such as surgical repair, embolization procedures, and conservative management [5].

## Case report

**Fig. 1:**
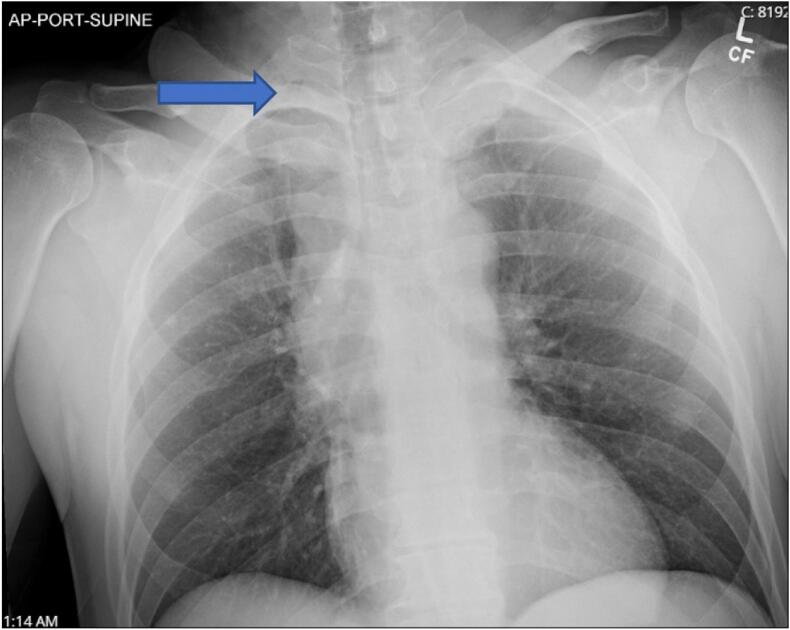
Chest X-ray on arrival to trauma center with right, apical pneumothorax.

**Fig. 2:**
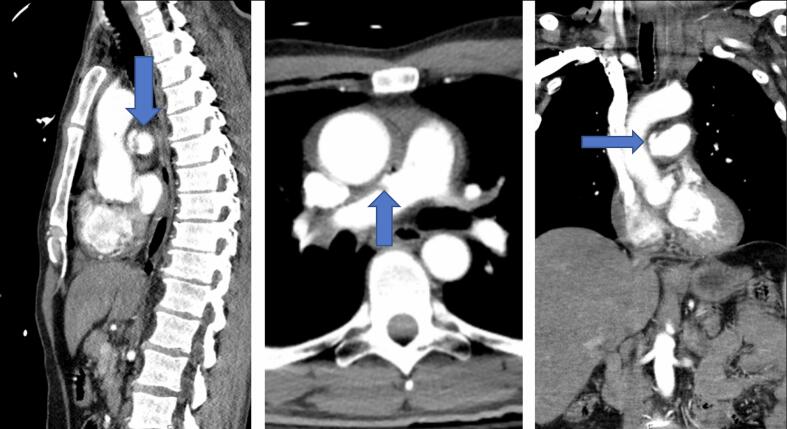
(A) Sagittal, (B) axial, and (C) coronal CT images demonstrating pseudoaneurysm in the proximal right pulmonary artery.

A 49-year-old man presented after a motor vehicle collision. He was the restrained driver, and there was a death in the vehicle. He was hemodynamically stable upon arrival with a Glasgow Coma Scale of 15 and complaining of chest and abdominal pain. An Advanced Trauma Life Support primary survey was within normal limits. He had diffuse abdominal tenderness and guarding with a well-defined seatbelt sign across the chest, abdomen, and left neck. Chest X-ray showed a small right apical pneumothorax (Fig. 1). Contrast enhanced CT scans of the neck, chest, abdomen, and pelvis were obtained demonstrating a proximal right PAP (Fig. 2), small volume hemopericardium, left first rib fracture, focal non-flow limiting dissection of left subclavian artery, widening of right sternoclavicular joint, disruption of right, second through fourth costochondral junctions, right anteromedial pneumothorax, right lower lobe pulmonary contusion, pneumomediastinum, hemoperitoneum with no solid organ injury, and stranding of central mesentery. Point of care cardiac ultrasound exam confirmed the finding of small hemopericardium with no tamponade physiology.

This patient was taken for an exploratory laparotomy due to a high suspicion for blunt hollow viscus injury. He was found to have a mid-jejunal perforation and bucket handle injury to the small bowel mesentery. This was managed with small bowel resection and stapled anastomosis with abdominal closure. Post-operatively the patient was admitted to the intensive care unit (ICU). For management of the right PAP, cardiac surgery was consulted before exploratory laparotomy and recommended a non-operative management strategy with an esmolol infusion for strict heart rate and blood pressure control. An echocardiogram was obtained the next day revealing no cardiac tamponade. CT angiography of the chest was done after 24 h which showed stable appearance of the right PAP and hemopericardium. Given this stability, the esmolol infusion was discontinued, and the patient was started on 10 mg of Amlodipine daily to maintain a systolic blood pressure less than 140 mmHg per the cardiac surgeon's recommendation. No oral beta blocker medication was administered as the patient's heart rate remained in a normal range throughout his hospitalization. A left subclavian artery angiogram was performed by the interventional radiologist on hospital day (HD) 10 due to neck swelling and this showed resolution of subclavian artery dissection and no pseudoaneurysm formation. After discussion with the cardiac surgeon, no further imaging was recommended in the inpatient setting. Patient was discharged home on HD 11 with Amlodipine 10 mg daily. Our intended follow up plan was to repeat imaging with CT angiogram of the chest in 3 months after discharge, and then every 6 months for 2 years to ensure stability of the pseudoaneurysm. We conducted this case report in compliance with the principles of the Declaration of Helsinki.

## Discussion

This report highlights the rare finding of a PAP after blunt trauma. True aneurysms are dilations of the vasculature containing all three histologic layers: tunica intima, tunica media, and tunica adventitia. Conversely, pseudoaneurysms are dilations comprised of an intramural hematoma within an outpouching of tunica media and adventitia. These aneurysms are more prone to rupture due to the very weak aneurysmal wall [4].

Presentation of these injuries may be asymptomatic and found incidentally on imaging. Conversely, dyspnea, cough, bloody effusion, life-threatening hemoptysis, and cardiogenic shock may also be present [3,[6], [7], [8]]. Early recognition of these symptoms in the appropriate setting improves outcome [6].

We selected a non-operative management strategy based on clinical and radiographic findings as well as expert recommendations from the cardiac surgery consultant. This patient had peritonitis, a seat belt sign, and signs of blunt hollow viscus injury on CT scan. Hence management of intraabdominal injury deserved the highest priority, and this was addressed first. Blunt PAP are exceedingly rare and no defined management guidelines currently exist. CT imaging of the patient in this case did not show a rupture of the PA and the amount of hemopericardium was small with no tamponade physiology. Unlike other arteries which are part of the systemic circulation, the PA is not exposed to high arterial pressure. The low-pressure system within the PA makes it possible for contained injuries to heal without overt risk of free rupture such as in an aortic injury. However, pseudoaneurysms are at risk of rupture and shear stress increases the risk of rupture. For this reason, we started the patient on an esmolol infusion to control heart rate and blood pressure. Heart rate control was more prudent in this case as the PA is not exposed to arterial pressures. Operative repair of the PA at this location would require a median sternotomy and cardiopulmonary bypass and as repeat CT scan showed stability of the pseudoaneurysm, operative intervention was not pursued.

Although standardized guidelines do not yet exist, there are several described management options for these injuries. The endovascular treatment for PA pseudoaneurysms involves occlusion by way of embolization, coiling, plugs or stents [1,6,9]. Additionally, cardiopulmonary bypass with surgical repair is an option, with primary anastomosis being utilized in certain cases [7,8]. Some traumatic PAPs have demonstrated spontaneous resolution [1]. For distal PAPs, some authors have described lobectomy for hemostasis with life threatening hemorrhage secondary to rupture. In one case report, a thoracotomy and left lower lobectomy was performed for a basal arterial trunk rupture [10].

In circumstances such as ours, non-operative management methods have proved to be successful, particularly if asymptomatic [3]. In asymptomatic patients, some PAPs resolve spontaneously which has been evidenced on repeat imaging [1].

Regardless of the method of management, follow up imaging guidelines have not been established. However, one report describes a patient who was followed with repeat imaging 3 months post injury and then every 6 months for 2 years. Imaging remained stable, and no further follow up was pursued [5].

Here we present the rare case of a PA pseudoaneurysm identified after blunt trauma that was managed with blood pressure and heart rate control. In the stable patient, impulse control and repeat imaging for stability may be adequate. Those pseudoaneurysms of large or unusual morphology may require a multidisciplinary approach with cardiothoracic surgery and interventional radiology to identify the optimal strategy for intervention.

## Declaration of competing interest

None. The authors declare no conflicts of interest but would like to recognize that the publication fees for this article were supported by the Kirk Kerkorian School of Medicine Library Open Article Fund.

## References

[1] Goel S., Kumar A., Gamanagatti S., Gupta A. (2013). Spontaneous resolution of post-traumatic pulmonary artery pseudoaneurysm: report of two cases. Lung India.

[2] Luiz L.G.R., Wynands E., Bourke M.E., Walley V.M. (1994). Catheter-induced pulmonary artery false aneurysm and rupture: case report and review. J. Cardiothorac. Vasc. Anesth..

[3] Lobato N., Reyes M., Lobob P., Benitoa M., Hernándeza G., De Graciac M., Dazaa S., Sendóna L. (2007). Pulmonary artery dissection and conservative medical management. Int. J. Cardiol..

[4] Restrepo C.S., Carswell A.P. (2012). Aneurysms and pseudoaneurysms of the pulmonary vasculature. Sem. Ultrasound CT MRI.

[5] Demondion P., Bellemare P., El-Hamamsy I. (2016). Conservative management of an intrapericardial contained rupture of the right pulmonary artery in blunt trauma: a good idea?. J. Thorac. Cardiovasc. Surg..

[6] McQueen A.S., Mitchell L., Muller M., MacGowan G., Corris P. (2008). Iatrogenic pulmonary artery pseudoaneurysm: images from different modalities. Thorax.

[7] Pereira S.J., Narrod J.A. (2009). Repair of right pulmonary artery transection after blunt trauma. Ann. Thorac. Surg..

[8] Collins M.P., Robinson G.C. (1989). Traumatic rupture of the pulmonary artery. Ann. Thorac. Surg..

[9] Sridhar S.K., Sadler D., McFadden S.D., Ball C.G., Kirkpatrick A.W. (2010). Percutaneous embolization of an angiographically inaccessible pulmonary artery pseudoaneurysm after blunt chest trauma: a case report and review of the literature. J. Trauma Injury Infect. Crit. Care.

[10] Riquet M., Dujon A., Lahsoune A., Molinier H. (1985). Traumatic rupture of the middle lobe bronchus and of the pulmonary artery. Based upon one case. J. Chir..

